# Cell and Gene Therapy for Anemia: Hematopoietic Stem Cells and Gene Editing

**DOI:** 10.3390/ijms22126275

**Published:** 2021-06-10

**Authors:** Dito Anurogo, Nova Yuli Prasetyo Budi, Mai-Huong Thi Ngo, Yen-Hua Huang, Jeanne Adiwinata Pawitan

**Affiliations:** 1 International PhD Program for Cell Therapy and Regeneration Medicine, College of Medicine, Taipei Medical University, Taipei 11031, Taiwan; d151109004@tmu.edu.tw (D.A.); d151109002@tmu.edu.tw (N.Y.P.B.); ntmhuong@hpmu.edu.vn (M.-H.T.N.); 2 Department of Biochemistry and Molecular Cell Biology, School of Medicine, College of Medicine, Taipei Medical University, Taipei 11031, Taiwan; 3 Faculty of Medicine and Health Sciences, Universitas Muhammadiyah Makassar, Makassar 90221, Indonesia; 4 Research Center of Cell Therapy and Regeneration Medicine, Taipei Medical University, Taipei 11031, Taiwan; 5 Graduate Institute of Medical Sciences, College of Medicine, Taipei Medical University, Taipei 11031, Taiwan; 6 Center for Reproductive Medicine, Taipei Medical University Hospital, Taipei 11031, Taiwan; 7 Comprehensive Cancer Center, Taipei Medical University, Taipei 11031, Taiwan; 8 Research Center of Cancer Translational Medicine, Taipei Medical University, Taipei 11031, Taiwan; 9 PhD Program for Translational Medicine, College of Medical Science and Technology, Taipei Medical University, Taipei 11031, Taiwan; 10 Department of Histology, Faculty of Medicine, Universitas Indonesia, Jakarta 10430, Indonesia; 11 Stem Cell Medical Technology Integrated Service Unit, Cipto Mangunkusumo Central Hospital, Faculty of Medicine, Universitas Indonesia, Jakarta 10430, Indonesia; 12 Stem Cell and Tissue Engineering Research Center, Indonesia Medical Education and Research Institute (IMERI), Faculty of Medicine, Universitas Indonesia, Jakarta 10430, Indonesia

**Keywords:** cell therapy, gene therapy, anemia, hematopoietic stem cells, gene editing

## Abstract

Hereditary anemia has various manifestations, such as sickle cell disease (SCD), Fanconi anemia, glucose-6-phosphate dehydrogenase deficiency (G6PDD), and thalassemia. The available management strategies for these disorders are still unsatisfactory and do not eliminate the main causes. As genetic aberrations are the main causes of all forms of hereditary anemia, the optimal approach involves repairing the defective gene, possibly through the transplantation of normal hematopoietic stem cells (HSCs) from a normal matching donor or through gene therapy approaches (either in vivo or ex vivo) to correct the patient’s HSCs. To clearly illustrate the importance of cell and gene therapy in hereditary anemia, this paper provides a review of the genetic aberration, epidemiology, clinical features, current management, and cell and gene therapy endeavors related to SCD, thalassemia, Fanconi anemia, and G6PDD. Moreover, we expound the future research direction of HSC derivation from induced pluripotent stem cells (iPSCs), strategies to edit HSCs, gene therapy risk mitigation, and their clinical perspectives. In conclusion, gene-corrected hematopoietic stem cell transplantation has promising outcomes for SCD, Fanconi anemia, and thalassemia, and it may overcome the limitation of the source of allogenic bone marrow transplantation.

## 1. Introduction

Hereditary anemia is caused by various hematological genetic disorders and has various manifestations, such as sickle cell disease (SCD), Fanconi anemia (FA), glucose-6-phosphate dehydrogenase (G6PD) deficiency, and thalassemia. In individuals with SCD, anemia is caused by sickle hemoglobin (Hb); under hypoxic conditions, sickle Hb is polymerized, leading to the formation of sickled-shaped red blood cells (RBCs) with a fragile membrane that are prone to hemolysis [1]. In FA, the genetic defect mainly causes cell cycle alterations and elevated cell death, which causes hematopoietic stem and progenitor cell (HSPC) depletion, followed by reduced blood cell formation. Reduced RBC formation causes aplastic anemia [2,3,4]. In G6PD deficiency, the pentose phosphate pathway (PPP) is disrupted, which is the only source of reduced nicotinamide adenine dinucleotide phosphate (NADPH). This disruption reduces the levels of NADPH, which protects RBCs from oxidative stress and protects the sulfhydryl groups of Hb from oxidation by oxygen radicals and hydrogen peroxide. Therefore, exposure to oxidative stress-inducing substances triggers the hemolysis of RBCs [5,6,7]. In thalassemia, the aforementioned genetic defect causes an abnormality in Hb production that causes anemia [8].

The available management strategies for these disorders are still unsatisfactory and mostly do not eliminate the main causes, namely genetic aberrations. Theoretically, the optimal approach for curing a hematological genetic disorder involves repairing the defective gene, possibly through gene therapy (GT), or by replacing the patient’s hematopoietic stem cells (HSCs) with normal HSCs from a healthy matching donor or with the patient’s own HSCs that have been genetically engineered.

This review addresses the prospect of cell therapy and GT for hereditary anemia by discussing SCD, thalassemia, FA, and G6PD deficiencies in terms of their genetic aberration, epidemiology, pathogenesis, clinical features, available management approaches and their limitations, and cell and GTs in animal models and clinical trials. In addition, we discuss future research directions related to HSC derivation from induced pluripotent stem cells (iPSCs), which are used in new genetic engineering techniques to eradicate the aforementioned genetic aberration, risk mitigation for engineered cell therapy, and related clinical perspectives.

## 2. SCD

SCD is a severe hereditary form of anemia that results from a single mutation in the sixth codon of the gene encoding the β-globin chain (from glutamic acid to valine) of the adult Hb tetramer (α2β2), which is prone to polymerization at low oxygen levels [9]. It is an autosomal recessive disorder caused by mutations in the Hb subunit β (*HBB*) gene, which encodes the β-globin subunit of HbA. It is characterized by the presence of RBCs that contain hemoglobin S (HbS) without additional normal HbA [10]. SCD may occur as sickle cell anemia (SCA), HbSC disease, and HbS-thalassemia. Genotypically, SCA has two sickle β (β^S^) alleles; HbSC disease has one sickle β allele and one β^C^ allele, which has another type of mutation; while HbS-thalassemia has one sickle β and one β null allele. Polymerized sickle Hb (HbS, α2βS2) leads to abnormalities in the RBC biconcave architecture and flexibility, resulting in crescent-shaped cells with an enhanced adherence to the vascular endothelium; these abnormal shaped RBCs obstruct blood flow, and are prone to hemolysis [11]. The terminology of SCD is used for all anemias caused by mutations in the *β-globin* gene. SCA is the most common form of SCD, representing 70% of SCD cases in African patients [12].

### 2.1. Epidemiology

An epidemiological study showed that 300,000 children with SCD are born every year worldwide [13]. Another study revealed that approximately 100,000 US nationals have SCD [14]. The areas with the highest prevalence of SCD are India, the Democratic Republic of Congo, and Nigeria [15]. In most areas of Africa, SCD affects approximately 1% of all births, and approximately 6–15% of affected children die before their fifth birthday. Approximately 50–90% of children develop SCD in Africa [16]. In Sudan, the prevalence of SCA ranges from 0.8% (Central Sudan) to 30.4% (Western Sudan). A study showed that the prevalence of SCA in Sudan is approximately 2–30.4% [1,17].

Mortality and morbidity caused by SCA in childhood are high. In the Unites States, the median survival is 48 years for women and 42 years for men [18], though advances in treatment options may increase survival, and a report from a London Hospital showed a median survival of 67 years [12]. A prospective cohort study in Kenya showed that children with SCD (58 per 1000 children/years) have a higher mortality than those without SCD (2.4 per 1000 children/years) [19].

### 2.2. Pathogenesis

The main pathogenic processes of SCD include HbS polymerization, dehydration, vaso-occlusion (VOC), hemolysis-mediated endothelial dysfunction, and sterile inflammation.

The polymerization of sickle Hb due to deoxygenation or hypoxic events is the most crucial and basic molecular pathogenic mechanism of SCD. This polymerization initiates the arrangement of contiguous strands that cause the formation of misshaped or crescent-shaped RBCs. This polymerization also initiates downstream events, such as alterations of the function and structure of the RBC membrane, deteriorations in vasoactivity, the unbalanced distribution of RBC volume, and increased RBC attachment to the endothelium [20,21].

The main etiology of the dehydration of sickled erythrocytes in SCD involves the activation of cytokines, endothelin 1 (ET-1), and prostaglandin E2 (PGE2) in the Gardos pathway [20]. The reticulocyte-rich fractions of sickled blood exhibit a high expression of K-Cl cotransporter isoform 2 (KCC2), which maintains optimal K^+^-Cl^−^ cotransport [22] and cation fluxes (Ca^2+^ ion entry), which are induced in a deoxygenation state [23]. In tissues with a high oxygen demand, the intraerythrocyte deoxygenation process causes hydrophobic motifs of deoxygenated HbS tetramers to be exposed [24].

In SCD, increased cell adhesion to the endothelium mediates VOC and biorheological disorders. Blood rheology is dependent on the viscosity of the plasma and hematocrit, as well as the deformability of erythrocytes. Because of decreased sickle erythrocyte deformability and persistent hemolysis owing to dehydration and Hb polymerization, increased plasma viscosity impedes the flow of blood through the venules of tissues with a high oxygen demand. Sickle erythrocytes have inadequate deformability and are sequestered in the microcirculation, causing transient VOC [25,26]. 

VOC can be triggered by multiple factors, such as stress, inflammation, ischemia, hemolysis, activated endothelial cells, increased plasma viscosity, and diminished blood flow [27]. E- and P-selectin, which are endothelial selectins, also play an important role in VOC. Random precapillary obstruction by a small number of dense sickle RBCs (SS-RBCs) also contributes to VOC. Epinephrine can activate intercellular adhesion molecule 1 (ICAM-4; an LW blood group glycoprotein), which is an RBC adhesion receptor that mediates SS-RBC adhesion to endothelial α-v-β-3 integrin. VOC enhances ischemia-reperfusion injury [28,29,30]. In addition, patrolling monocytes are reduced in recent VOC episodes. Patrolling monocytes, which scavenge debris and damaged cells from the vasculature, have higher levels of the anti-inflammatory heme oxygenase 1 (HO-1) enzyme, which degrades heme. Patrolling monocytes expressing HO-1 protect the SCD vasculature from ongoing hemolytic insult and vaso-occlusion [31].

Endothelial dysfunction is a pathognomonic factor related to SCD, and is caused by the upregulation of P- and E-selectin, ICAM-1, vascular cell adhesion molecule-1 (VCAM-1), and major leukocyte chemoattractants (i.e., keratinocyte-derived chemokine in mice or interleukin-8 in humans) on endothelial cells [24]. Repeated vascular damage resulting from VOC may cause endothelial cell dysfunction through a mechanism resembling ischemia/reperfusion injury [28].

Together with heme and iron, endothelial cells are key players and have an important role in SCD oxidative damage through several actions. First, they are involved in the formation of robust oxidizing species, such as ferryl-Hb and •OH, through the H_2_O_2_-dependent Fenton reaction. Second, increased platelet adhesion may activate endothelial cells. Third, increased inflammatory cytokine production (interleukin (IL)-1-β, IL-6, and IL-18, and tumor necrosis factor (TNF)-α), inflammasome activation, and the stimulation of Toll-like receptor-4 (TLR4) in endothelial cells occur through NF-κB-linked pathways. In addition, there is reduced anti-inflammatory cytokine IL-10 that is correlated with the frequency and severity of VOC [32]. Fourth, activated neutrophils may influence endothelial cells and serve as neutrophil extracellular traps (NETs) for RBCs and platelets. Fifth, the expression of adhesion molecules such as P-selectin, E-selectin, ICAM-1, and VCAM-1 is increased; these are all markers of endothelial dysfunction, and can serve as receptors for leukocytes (monocytes, neutrophils, and lymphocytes). Sixth, blood coagulation is triggered by the release of intraluminal tissue factor from endothelial cells—the tissue factor then binds to factor VIIa, promoting the coagulation cascade [33].

The association of sterile inflammation with SCD-related morbidity suggests that anti-inflammatory paradigms are treatments for SCD [34]. Sterile inflammation may be promoted by damage-associated molecular patterns, such as heme, Hsp70, ATP, cyclophilin A, mtDNA, HMGB1, extracellular DNA, and S100A8, which may play important roles in SCD inflammatory mechanisms [35].

CXCL1 is a fundamental inflammatory biomarker of VOC [36]. The administration of CXCL1 exogenously is adequate to activate VOC, and the suppression of the CXCL1 receptor (namely CXCR2) may prevent VOC development because of the hemolytic transfusion reaction. The direct suppression of this pathway may be a novel therapy for VOC [37].

SCD features a vicious circle between inflammation and abnormal RBC rheology [38], which modulates clinical severity in patients. Chronic inflammation leads to organ dysfunction in SCD patients. High activation of coagulation factors, endothelium, monocytes, neutrophils, and platelets are key factors in this vicious cycle. Different strategies have been used to determine the effect of these factors in SCD patients [39].

### 2.3. Clinical Features of SCD

SCD or SCA has various clinical manifestations, such as asplenia, severe infection, episodes of VOC, neurological changes, priapism, stroke, acute chest syndrome, renal failure, pain crisis, and pulmonary hypertension [40]. On the basis of a retrospective study in children with SCA in Rio de Janeiro, their clinical features are infection (27–35%), hemolytic crisis (25%), splenic sequestration (17–21%), painful events (12–16%), and hand–foot syndrome (12–16%) [41].

### 2.4. Current Management of SCD

The current management approaches for SCD are hematopoietic stem cell transplantation (HSCT), various drugs, and other strategies [42].

#### 2.4.1. HSCT

Allogeneic human leukocyte antigen (HLA)-matched HSCT is the only curative treatment for SCD. It is curative in 90–95% of SCD patients [43]. Matched sibling donor (MSD) HSCT is a curative strategy for all patients aged ≤ 5 years old as well as older pediatric patients older between 5 to 18 years old presenting with SCD-derived complications. When performed in time, HSCT can establish donor-derived erythropoiesis and stabilize or even restore the function of affected organs in patients with SCD. Haploidentical-bone marrow transplantation (BMT) with posttransplant cyclophosphamide (PTCy) has emerged as a viable and safe option for HSCT in patients with severe SCD, and it results in a lower rate of graft versus host disease (GVHD), but improvements are required to increase the low engraftment rate. When ex vivo-expanded, partially mismatched unrelated cord blood (UCB) grafts are used, rapid donor cell engraftment may lead to a reduction in SCD severity. The risks of infections and severe GVHD must be minimized [24,33,34,35].

Following HSCT, SCD patients can develop mixed chimerism (MC), that is, the co-existence of host- and donor-derived cells; however, the clinical control of SCD can still be ensured. In an SCD patient with MC, the expression of the apoptotic regulator Fas was significantly higher in the recipient erythroblasts and RBCs than in the donor erythroblasts and RBCs, suggesting that SCD “ineffective” erythroid cells undergo apoptosis, whereas donor cells have a survival advantage. Stable donor chimerism greater than 25% is associated with the resolution of SCD-related symptoms [36,37,38].

Pilot studies of BMT for the management of young symptomatic SCD patients showed a low transplantation-related mortality and resolution of the underlying disease. Two-thirds of BMT recipients remained VOC-free over 2 years of follow-up, but transplant-related complications, including GvHD, occurred with a high frequency. Currently, 35 clinical trials registered at ClinicalTrials.gov (accessed on 1 April 2021) are studying allogeneic BMT in patients with SCD [39,40,41].

To reduce the graft failure rate of T-cell-replete haploidentical transplantation, pretransplant immune and myelosuppression (PTIS), which involves the use of cyclophosphamide, dexamethasone, and fludarabine, followed by the augmented John Hopkins protocol with thiotepa-based conditioning and plerixafor-based mobilization in healthy donors, is effective and safe for treating patients with SCD, with minimal risk of graft failure and low GVHD rates [44].

#### 2.4.2. SCD Management Approaches Other Than HSCT

The current drug management approaches related to SCD are classified into four categories based on their target. The first category is of drug compounds that target erythrocyte rheology, reduce polymerization, and improve cellular hydration. Novel approaches for inhibiting HbS polymerization may be achieved through several mechanisms, such as activating HbF synthesis, increasing oxygen (O_2_) affinity, inhibiting intermolecular connections in sickle strands in RBCs by decreasing the concentration of 2,3-diphosphoglycerate, and decreasing the intracellular Hb concentration. A decline in the intracellular Hb concentration generally occurs in patients who have developed iron deficiency [23]. Examples of these drugs are hydroxyurea, senicapoc, metformin, Aes-103, and voxelotor. The second category contains drug compounds that reduce VOC and cellular adhesion, such as hydroxyurea, crizanlizumab, rivipansel, Ig (intravenous), tinzaparin, dalteparin, sevuparin, eptifibatide, NKTT120, ticagrelor, and prasugrel. The third category includes drug compounds that improve endothelial dysfunction, including hydroxyurea, l-glutamine, haptoglobin, arginine, oral or intravenous nitrite, inhaled nitric oxide, and antioxidants. The fourth category contains drug compounds that improve sterile inflammation, including hemopexin/haptoglobin, MP4CO, various antioxidants, DNAse-1, canakinumab, montelukast, simvastatin, and anakinra, as well as TLR4 inhibition. Drugs that are approved by US Food and Drug Administration (FDA) for SCD are hydroxyurea, l-glutamine, and crizanlizumab [45]. Despite the availability of standard therapies and novel management approaches (i.e., hydroxyurea, blood transfusion, hydration, and pain-relieving medicines), patients continue to experience long-term complications from the disease [46]. In addition, the safety and efficacy of related medicines are still under investigation in clinical trials (Table 1).

Hydroxyurea is a ribonucleotide reductase inhibitor [12], and is regarded as a drug of choice for improving endothelial dysfunction (as a nitric oxide donor) and red cell rheology. Beneficial effects include the induction of fetal hemoglobin (HbF) for inhibiting erythrocyte adhesion, conceivably ensuring nitric oxide donor bioavailability; myelosuppression (resulting in the reduced availability of leukocytes, reticulocytes, and platelets) and reduced frequency of acute pain; and alleviation of acute chest syndrome, reduced hospital admissions, and reduced blood transfusions. It may also protect against progressive organ damage, including nephropathy [47,53]. Moreover, clinical trials in infants and toddlers have shown very promising results [12]. However, as the most prominent side effect of hydroxyurea is in adult men with SCD, fertility problems are still an important concern worldwide [54].

#### 2.4.3. GT Approaches for SCD

A GT approach toward SCD was developed in which HbF production is permanently enhanced using various vectors. One vector is the lentiviral vector (LV), which induces the overproduction of γ-globin. LVs have been proven to efficiently transmit complex globin expression cassettes containing transcriptional regulatory sequences from the β-globin locus control region, which are required for a high expression. A similar approach has been used in studies involving mouse models of SCD. The overexpression of a β-globin polypeptide containing specific point mutations was designed to optimize the antisickling activity and led to the improvement of SCD in two models. In BERK SCD mice, efficient γ-globin LV gene transfer resulted in steady-state HSCs. Moreover, a clinically relevant forward-oriented β-globin–expressing vector, with six-fold higher vector titers and a four- to ten-fold higher transduction efficiency, was tested for the long-term repopulation of hematopoietic cells in humanized mice and rhesus macaques. The insertion of the Rev response element enabled the retention of intron 2, and β-globin production was observed in macaques transplanted with human SCD CD34+ cells. Another vector is the adenoviral vector. In vivo HSC GT using the bimodular HDAd5/35++ vector cured SCD in a mouse model. Compared with HDAd vectors with either γ-globin addition or CRISPR-Cas9 reactivation units, in vivo HSC transduction of CD46/Townes mice with the HDAd combo resulted in significantly higher γ-globin production in RBCs, which reached 30% of the production of adult human α and βS chains, and resulted in complete phenotypic correction for SCD. Some clinical trials (NCT02247843, NCT02140554, and NCT02186418) are ongoing and are using lentiviral strategies to induce HbF [42,44,45,46].

Another approach is the knock down of BCL11A, which is a repressor of γ-globin expression and HbF production, in adult erythrocytes. The inhibition of BCL11A was effective for inducing HbF. This is initial proof that shmiR-based gene knockdown results in an advantageous risk–benefit profile in SCD. Moreover, inactivation of the transcription factor BCL11A in a humanized and transgenic SCD mouse model resulted in the adjustment of the pathologic and hematologic defect that was related to SCD [55].

#### 2.4.4. Engineered Stem Cell Approach for SCD

The engineered stem cell approach for SCD consists of three processes. The first is harvesting autologous HSPCs from bone marrow or peripheral blood. The second is genetically modifying sickle HSPCs by gene editing. The third approach involves transplanting the gene corrected HSPCs back into the patient after chemotherapy conditioning. Gene-modified HSPC graft repopulates the hematopoietic stem compartment, producing genetically corrected RBC progeny. Sickle HSPCs can be genetically modified through zinc finger nucleosomes (ZFNs), transcription activator-like effector nucleases (TALENs), or clustered regularly interspaced short palindromic repeats/CRISPR-associated nuclease 9 (CRISPR/Cas9) techniques (NCT03745287) [47,48,49,50].

## 3. FA

FA is a rare X-linked autosomal genetic disease that causes hereditary bone marrow failure (BMF) syndrome, and it is characterized by pancytopenia, progressive BMF, congenital malformations or abnormalities, sensitivity to cross-linking agents, cancer predisposition, and increased cancer risk during early adulthood [4,56]. This inherited disease, which was first reported in 1920s by Guido Fanconi, is associated with a median lifespan of 33 years [57].

### 3.1. Epidemiology of FA

FA is an uncommon type of anemia. The incidence of FA is approximately 1 in 136,000 newborns and 1 in 360,000 live births. Its incidence varies from 1 in 100,000 to 250,000 births, and 1 in 181 patients is a carrier. Data and registries from Europe show that the prevalence of FA is approximately 4–7 per million live births. A diagnosis of FA is difficult because of the wide range of symptoms [56,58].

### 3.2. Pathogenesis of FA

FA is caused by germline mutations in any of 22 genes (*FANCA*, *FANCB*, *FANCC*, *FANCD1/BRCA2*, *FANCD2*, *FANCE*, *FANCF*, *FANCG*, *FANCI*, *FANCJ/BRIP1*, *FANCL*, *FANCM*, *FANCN/PALB2*, *FANCO/RAD51C*, *FANCP/SLX4*, *FANCQ/ERCC4/XPF*, *FANCR/RAD51*, *FANCS/BRCA1*, *FANCT/UBE2T*, *FANCU/XRCC2*, *FANCV/REV7*, and *FANCW/RFWD3*) [59]. These FA gene products disrupt a cellular pathway (FA pathway) that is mainly involved in DNA repair mechanisms and is responsible for the resolution of DNA interstrand cross-links (ICLs) [60,61,62]. ICLs are a type of DNA damage and are primarily caused by cytotoxic chemotherapeutic agents. DNA damage in HSCs is caused by reactive aldehydes, suggesting that they play a fundamental role in FA and genome instability [4].

The FA pathway is a biochemical network that functions in collaboration with DNA replication, DNA repair, and other biocellular processes [63]. The fundamental function of the FA pathway is to activate proteins for the repair of ICLs for alleviating replication stress and genomic instability [64]. In FA, a genetic mutation occurs in *FANCV/REV7* that has multiple activities [65]. The hallmarks of FA cells are cell cycle alterations, chromosome and genomic instability, a DNA repair defect in relation to sensitivity to aldehyde and oxygen, defects in cell reprogramming, elevated cell death, and overactivity of the tumor suppressor p53 [2,3,4]. The overactive p53 causes HSPC depletion in the bone marrow of FA patients [2], which is the cause of aplastic anemia.

### 3.3. Clinical Features of FA

FA has several clinical features, namely aplastic anemia, congenital abnormalities (skin hyperpigmentation such as café au lait spots, short stature, and thumb and radius deformities or abnormalities), and predisposition to epithelial cancers and acute myelogenous leukemia [65,66,67]. Other classic clinical manifestations are major diagnostic keys, such as Fanconi facies, microcephaly (small head size), microphthalmia, developmental delay, structural abnormalities (renal, cardiac, genitourinary, and other malformations), and radial ray defects. FA can be diagnosed in adulthood and may occur in persons without congenital defects [68].

The development of macrocytosis and the induction of HbF usually occur, but their nonappearance does not exclude the disease. FA should be suspected in all children and young adults with one or more of the following symptoms: aplastic anemia, hypoplastic anemia, cytopenia, acute myeloid leukemia, myelodysplastic syndrome (MDS), epithelial malignancies, unexplained macrocytosis, or characteristic physical anomalies. An association exists between the first adverse outcome in the form of early-onset BMF and the number of major congenital anomalies [69,70,71].

The fundamental pathognomonic factors of FA can be classified into several types: bone marrow disorders, abnormalities in embryo development (e.g., microcephaly and birth defects), cancer predisposition (such as leukemia and solid tumors), and endocrine and reproductive defects [72].

### 3.4. Current Management of FA

A life-threatening complication of FA is BMF. Therefore, the management of BMF is of primary importance [70]. Stem cell transplantation (SCT) is the optimal choice for inducing normal hematopoiesis. Patients with severe pancytopenia (Hb ≤ 8 g/dL, absolute neutrophil count < 1000/μL, or platelet count ≤ 40–50,000/μL) who are otherwise healthy and have an available HLA-MSD are ideal candidates for HSCT [73,74].

The optimal supportive management strategies for FA are infection prophylaxis and the transfusion of packed erythrocytes (RBCs) and platelets. These treatments have immediate effects. Because of GVHD and alloimmunization, RBC transfusion from non-HLA-matching family members should be avoided. In patients with hematopoietic cell transplantation, the transfusion of a high volume of units has poor outcomes. In patients with leukopenia, treatment with granulocyte-colony-stimulating factor generates an adequate response, but this response is limited in patients with an absolute neutrophil count less than 200/mL [4,58].

#### 3.4.1. GT Approaches for FA

GT approaches focusing on correcting the genetic defect in a patient’s HSCs are used to treat FA-associated BMF [75]. Functional copies of the FA gene are incorporated into a viral vector for in vivo gene insertion into the patient’s defective genome. Various vectors may be used in GT for FA, such as adenoviruses and retroviruses. Retroviruses have the potential to exchange defective FA genes, such as *FANCC*, with a normal one. With the current advances, LVs, which are retroviruses, have favorable safety profiles with optimized transcriptional levels for improving hematopoietic progenitors in FA, whereas γ-retroviruses are correlated with genotoxicity [66]. The use of LVs in GT for FA management is supported by the favorable results of clinical trials involving β-thalassemia, adrenoleukodystrophy (ALD), Wiskott–Aldrich syndrome, and metachromatic leukodystrophy [76].

#### 3.4.2. Engineered Stem Cell Approach for FA

Gene editing of patients’ cells has been revealed to be the optimal solution for FA. As a therapeutic application, gene editing might correct mutated genes in patients’ HSCs. Nevertheless, intrinsic FA DNA repair defects might render gene editing impractical. Gene editing was demonstrated to adequately restore FANCF function through error-prone end joining, which resulted in a 27% increase in cell survival under treatment with mitomycin C (MMC). After the repair of double- or single-strand breaks through CRISPR/Cas9-mediated correction, templated gene correction could be accomplished. Even though the efficiency of templated gene editing was low (≤6%), the proliferation of corrected FA embryonic stem cells occurred in preference to noncorrected cells, even without genotoxic stress. In the study, the use of Cas9 nickase ensured the absence of off-target mutagenesis and monoallelic gene editing [77].

#### 3.4.3. GT and Engineered Stem Cell Clinical Trials on FA

Several clinical trials of gene therapies were conducted for the development of FA management approaches. In a clinical trial for the treatment of type C FA, retroviral vectors were used to transfer a normal FACC gene to primary hematopoietic cells and FA(C) lymphoid cell lines. The introduction of the normal FACC gene into CD34+ progenitors distinctly increased their development in the presence and absence of MMC, whereas mutant FACC gene-containing CD34+ progenitors were destroyed. This clinical trial was registered as NCT00001399.

Another clinical trial (NCT01331018) evaluated the efficacy and toxicity of the infusion of gene-corrected cells for FA patients. The infusion of autologous corrected blood stem cells potentially enhanced the blood count in FA patients. A third clinical trial determined the efficacy of genetically engineered hematopoietic cell-based therapy for patients with subtype A FA (FA-A). Enriched CD34+ HSCs were transduced ex vivo with a therapeutic normal FA gene harboring LV, and were then infused back to patients intravenously without any preceding conditioning. This study was registered as NCT04248439.

## 4. G6PD Deficiency

G6PD deficiency is an X-linked genetic disorder and is the most common X-linked enzymopathy; exposure to certain drugs, foods, stressors, or infection can lead to hemolytic anemia and multiorgan failure and then mortality. Therefore, the optimal management for G6PD deficiency is the avoidance of substances that can trigger hemolysis, although this strategy is not feasible in parasitic endemic areas in certain regions. G6PD deficiency affects more than 400 million people worldwide [78,79,80]. A deficiency that occurs secondary to the mutation of the G6PD gene on the X chromosome was discovered in the 1950s, when American soldiers experienced acute hemolytic anemia due to antimalarial treatment; this deficiency is known as primaquine sensitivity syndrome [81,82].

G6PD deficiency is induced by a mutation in the *G6PD* gene (OMIM 305900) located on the long arm of the X chromosome (Xq28). The *G6PD* gene comprises 13 exons, spanning 18 kb, and encodes the 515-amino acid G6PD enzyme (OMIM 305900) [83]. The protein-coding region is composed of 12 segments, varying in length from 12 to 236 bp. An intron is present in the 5′ untranslated region. The major 5′ end of mature G6PD mRNA is located 177 bp upstream of the translation-initiating codon in several cell lines. Comparison of the promoter region of G6PD and 10 other housekeeping enzyme genes proved the existence of these ordinary features. Moreover, a conserved 25-bp sequence was detected promptly downstream of a TATA box in eight patients [84].

As hemolysis occurs upon exposure to certain substances, tests are required to identify the substances that may trigger hemolysis in patients with G6PD deficiency. An interventional study was conducted to evaluate a new and safe testing method for identifying the hemolytic potential of drugs in 14 patients with G6PD deficiency (NCT00076323). Another study assessed the incidence of hemolysis and the safety and efficacy of tafenoquine (SB-252263, WR238605) versus primaquine in 251 patients with G6PD deficiency who had *Plasmodium vivax* (*P. vivax*) malaria (NCT02216123). As G6PD is due to an abnormality in the X chromosome, men are usually more affected. A study evaluated the safety of primaquine combined with dihydroartemisinin-piperaquine in 61 male patients with G6PD deficiency in the Gambia (SAFEPRIM-II), but the study was terminated before final cohort recruitment because of financial and logistic reasons (NCT02654730). Furthermore, a study on the safety and efficacy of different regimens of primaquine for *P. vivax* malaria in 104 patients with G6PD deficiency is ongoing and is scheduled to end in July 2022 (NCT03529396).

### 4.1. Epidemiology

It has been predicted that 7.5% of the population carries G6PD mutations. The prevalence of G6PD deficiency differs by region. Its prevalence is more than 30% in African regions and approximately 0.1% in Japan and certain European countries. The incidence is high in Taiwan and the southeastern region of China [85]. A study showed that in the Thai population, the prevalence of G6PD deficiency was 3–18% [86]. Another study on a homogeneous population in Nigeria showed that the prevalence of G6PD deficiency was 23.9% in men and 4.6% in women [87].

### 4.2. Pathophysiology

G6PD converts glucose-6-phosphate into 6-phosphogluconolactone in the first step of PPP; then, glucose is modified into pentose sugars for nucleic acid synthesis. G6PD catalyzes nicotinamide adenine dinucleotide phosphate (NADP+) to its reduced form (NADPH). NADPH protects cells from oxidative stress. Additionally, NADPH is involved in the regeneration of the reduced form of glutathione, which protects the sulfhydryl groups of Hb (which are susceptible to oxidation by oxygen radicals and hydrogen peroxide). Although G6PD is present in every cell, G6PD deficiency results in hematologic defects, because PPP is the only pathway producing NADPH in erythrocytes [88,89]. Erythrocytes are more susceptible than other cells to destruction from oxidative stress, because NADPH is only produced through PPP in erythrocytes. In affected erythrocytes, the G6PD activity is lower than that in other cells. Normal erythrocytes that are not under oxidative stress typically exhibit a G6PD activity at nearly 2% of the total capacity. Even under a considerably diminished enzyme activity, few or no clinical features might present when no exposure to hemolysis-triggering conditions has occurred [88,89].

### 4.3. Clinical Features

Most patients with G6PD deficiency are asymptomatic. However, they may have clinical manifestations such as favism, neonatal jaundice, or chronic nonspherocytic hemolytic anemia. Common symptoms of acute hemolysis, which is associated with G6PD deficiency, include fatigue, anemia, pain (abdominal pain or back pain), hemoglobinuria, and jaundice (decrease in reticulocyte count to <50% of normal values) [89,90,91].

### 4.4. Diagnosis

G6PD deficiency can be diagnosed through various procedures, such as through the detection of the enzyme activity through spectrophotometry, a UV fluorescence visualization test, a formazan-based spot test, and biosensors and test strips. Furthermore, molecular analysis can be utilized to discover suspected mutations of the G6PD encoding gene.

Globally, 217 mutations have been reported in the *G6PD* gene [92]. Mutations in the *G6PD* gene may occur at various sites. Through the use of gene sequencing and polymerase chain reaction, various mutations were revealed in children of Han Chinese ethnicity, such as c.202G>A (in exon 4), c.406C>T (in exon 5), c.487G>A and c.493A>G (in exon 6), c.697G>C (in exon 7), c.1311C>T (in exon 11), c.1376G>T and c.1388G>A (in exon 12), 1365-13T>C (in intron 11), and 493A>G (in intron 6) [93]. Molecular analysis through direct DNA sequencing of the *G6PD* gene in 102 Thai pediatric patients with G6PD deficiency revealed 12 missense mutations, namely G6PD Viangchan (871G>A), G6PD Canton (1376G>T), G6PD Kaiping (1388G>A), G6PD Mahidol (487G>A), G6PD Quing Yan (392G>T), G6PD Coimbra (592C>T), G6PD Union (1360C>T), G6PD Songklanagarind (196T>A), G6PD Valladolid (406C>T), G6PD Aures (143C>T), G6PD Chinese-5 (1024C>T), and G6PD Mediterranean (563C>T) [94].

G6PD deficiency diagnosis can be made in women homozygous for *G6PD* gene mutations [89,95]. Moreover, women heterozygous for *G6PD* gene mutations may give birth to hemizygous male infants who could develop hemolytic crisis upon inadvertent exposure to oxidative stress, which could induce kernicterus, hyperbilirubinemia, and even death [96].

### 4.5. Management of G6PD Deficiency

No effective treatment exists for G6PD deficiency [89]. Its management primarily consists of the cessation of triggers and the provision of supportive care. The utilization of antioxidants (such as selenium and vitamin E) is inadequate for managing G6PD deficiency [88,97]. Pegloticase, rasburicase, and tafenoquine are contraindicated in persons with G6PD deficiency [98,99,100].

Small molecules are a promising therapeutic approach for G6PD deficiency. AG1 (2,2′-disulfanediylbis(*N*-(2-(1*H*-indol-3-yl)ethyl)ethan-1-amine) is a tiny molecule that increases the stability of the wild-type gene, Canton mutant, and various other ordinary G6PD mutants. In human erythrocytes, AG1 decreases diamide- or chloroquine-induced oxidative stress. Hwang et al. (2018) identified AG1 through high-throughput screening. AG1 mediated the increased activation of the G6PD enzyme. AG1 decreased ROS levels and hemolysis and increased GSH levels in both healthy erythrocytes exposed to chloroquine and erythrocytes stored for 28 days that developed storage lesions [79,97].

Until recently, no report on genetic engineering or engineered stem cell approaches for G6PD deficiency was available. However, when the underlying mutation is known, the mutation can be corrected using GT or through the correction of patients’ HSC, and the subsequent autologous transfusion of the corrected HSCs for severe G6PD deficiency, especially in parasitic endemic areas where exposure to hemolysis-triggering agents, is highly possible.

## 5. Thalassemia

Thalassemia is an autosomal recessive disease that causes inherited defects in the production of Hb. Approximately 1–5% of the global population are carriers for a thalassemia genetic mutation [101]. Each year, more than 50,000 patients develop severe thalassemia worldwide, such as β-thalassemia major (BTM) and HbE β-thalassemia [102].

### 5.1. Epidemiology

The prevalence of β-thalassemia is approximately 288,000 globally. β-thalassemia is considered an enigmatic disease in the European Union and United States [103]. In the Eastern Mediterranean region, the number of infants born with β-thalassemia each year is the highest in Pakistan [104]. In Thailand, the overall prevalence of thalassemia carriers among six main hill tribe populations was 9.8% (117 of 1200) [105].

### 5.2. Classification

Thalassemia can be classified into transfusion-dependent thalassemia (TDT) and non-transfusion dependent thalassemia (NTDT) based on the requirement of regular blood transfusions. The clinical heterogeneity of thalassemia is presented in Table 2. The spectrum ranges from asymptomatic (or with mild anemia (thalassemia minor)) thalassemia carriers, to symptomatic (mild, moderate, or severe) thalassemia (TDT or NTDT).

A comprehensive understanding of the pathophysiology of thalassemia has led to the development of new therapeutic strategies [106].

### 5.3. Current Management of Thalassemia

Many strategies are available for the treatment of thalassemia, including blood transfusion, iron chelation therapy (ICT), drugs such as hydroxyurea, HSCT, GT, and gene editing of patients’ stem cells.

#### 5.3.1. Blood Transfusion

Blood transfusion can maintain HbF levels and modulate the severity of β-thalassemia, but it usually causes iron overload. Therefore, ICT is required. Although a universal transfusion protocol for TDT is not feasible, long-term transfusion therapy for individuals with thalassemia is based on common principles. Early initiation of RBC transfusions, avoidance of splenectomy, and extended matching for Rh and K antigens can reduce the incidence of alloimmunization in patients with BTM. Data from registries show that 64–89% of patients with homozygous or compound heterozygous β-globin thalassemia mutations required regular transfusions at intervals between 2 and 4 weeks. The transfusion-associated complications reported included iron overload, transfusion reactions, alloimmunization, and infections [49,107].

#### 5.3.2. ICT

Appropriate ICT may support the regular blood transfusions required by patients with β-thalassemia major (BTM) throughout their life. ICT should be started within a year of initiating regular blood transfusions. ICT improves overall survival and decreases the risk of complications related to iron overload, such as heart disease, short stature, and endocrine abnormalities. The timely initiation of iron chelation, appropriate dosing, and high compliance are fundamental to achieving the optimal control of iron overload [108,109].

#### 5.3.3. Drugs for Increasing HbF Levels

Hydroxyurea is a cost-effective drug that can increase HbF levels and enhance total Hb levels. It is currently approved for the treatment of non-transfusion-dependent β-thalassemia. The mechanism of action of hydroxyurea involves the activation of the *γ-globin* gene, which increases Hb F synthesis [110,111].

#### 5.3.4. HSCT

HSC transplantation can be regarded as a once in a lifetime therapy for the production of normal Hb. Before transplantation, defective stem cells must be destroyed, and an HLA-matched donor is required. Otherwise, immunosuppression is necessary. Allogeneic SCT is curative for patients with severe thalassemia. Busulfan combined with cyclophosphamide may be used as a nonmyeloablative conditioning regimen prior to SCT to get a better clinical outcome. In this nonmyeloablative regimen, the dose of busulfan and cyclophosphamide were adjusted to reduce the host HSC, but did not cause deleterious side effects, and thus provided conditioning for the transplant to engraft [112,113].

#### 5.3.5. GT Approaches for Thalassemia

GT in vivo approaches involving the use of globin LVs to inhibit the *BCL11A* gene are currently under investigation. In addition, the erythroid-specific knock down of BCL11A delivered by a lentiviral-encoded, microRNA-adapted short hairpin RNA molecule has been shown to reactivate the *γ-globin* gene and is in early clinical development. GT, called living medicine, promotes the normal differentiation of erythropoietic cells. However, it may lead to tumor formation and may exhibit viral toxicity [101,114,115,116].

An additional thalassemia management approach is the reactivation of γ-globin, which can replace defective β-globin, through the knock down of proteins that prevent HbF expression, thus promoting HbF formation [117]. EIF2AK1, which is also recognized as heme-regulated inhibitor HRI (HRI), has a fundamental role in the translation of proteins that prevent the expression of HbF [118]. HRI is an erythrocyte kinase enzyme that inhibits the translation of HbF [119].

A peptide nucleic acid (PNA)-based gene editing method with a single in utero dose of nanoparticles containing donor DNA and PNA alleviated β-thalassemia in the postnatal period and in an experimental (adult) mouse model. The breakthrough of PNA technologies into gene editing has enabled constructional biochemistry, disease phenotype improvement, and clinical protein remodeling; thus, this is a prospective remedial therapy toward monogenic disorders [120,121,122].

Clinical trials were conducted on gene editing of autologous HSPCs through LV-mediated normal globin gene transfer for β-thalassemia, such as the LG001 study (phase I/II: NCT01639690, phase I: NCT01745120), HGB-204 phase I/II (NCT02151526), HGB-205 phase I/II (NCT02453477), TIGET-BTHAL phase I/II (NCT02906202), HGB-207 phase III (NCT03207009), and HGB-212 phase III (NCT03207009). Phase I/II trials were completed, and several treatments were reported to be safe and effective, but phase III trials are ongoing. Another gene editing approach for autologous HSC, which involves using Sangamo’s ZFN to disrupt the *BCL11A* gene enhancer that suppresses the fetal Hb expression in RBCs, is under investigation in an ongoing trial (NCT03432364, ST-400-01 (phase I/II)). Furthermore, gene editing of autologous HSPCs using CRISPR-Cas9 is under investigation in an ongoing trial (NCT03655678, CTX001-111 (phase I/II)). The advances in gene editing and Bcl11a-mediated *γ-globin* gene silencing are the foundations for another GT approach with the purpose of restoring the production of HbF [123]. These clinical trials established the tolerability and practicability of GT for β-thalassemia; they also revealed its safety and efficacy [124,125,126]. However, two major obstacles to the safety and efficacy of GT for β-thalassemia remain. The first obstacle is the compilation of adequate numbers of HSCs (namely CD34+ cells) cautiously. The second obstacle is the transduction of patients’ CD34+ HSCs at therapeutic levels [124].

#### 5.3.6. Engineered Stem Cell Approaches for Thalassemia

Advancements in the ex vivo genome editing of autologous HSCs have led to genetically corrected iPSC-derived HSCs that can undergo differentiation in vivo and produce human β-globin in a mouse model. Mice that received transplants lived for more than 10 weeks without tumor formation. These results have important implications for the future personalized treatment of β-thalassemia, as it is a lifetime therapy, and no immunosuppression is required. However, gene editing may exhibit an off-target activity. Although preliminary results support further experimental testing of CRISPR-Cas9 gene editing approaches, these off-target activities should be addressed [115,116].

## 6. Advantages and Disadvantages of GT and Engineered Stem Cell Approaches

The advantage of GT-based correction of hematopoietic cells, which can be conducted in vivo or ex vivo, is that the treatment effects will be durable for life when successful. However, although GT and cell reprogramming are promising treatments, several limitations present challenges for regenerative medicine, especially for treating FA or other hereditary anemias. In vivo GT using normal gene insertion through a vector may be promising, but whether the vector inserted the gene in the correct site is uncertain and is challenging to determine. Ex vivo genetically engineered HSCs can be checked before administration. However, some technical difficulties occur in terms of collecting sufficient patient cells for gene correction and autologous infusion. One solution is to generate iPSC-derived hematopoietic cells. Mouse model studies showed that advanced technologies for HSCT are restricted by difficulties in repopulating hematopoietic cells in transplanted animals with either mouse or human iPSC-derived hematopoietic cells. Although some experiments have demonstrated the feasibility of inducing hematopoiesis by utilizing reprogrammed cells in mice, further advancements are necessary for transplantation in patients with monogenic diseases affecting the hematopoietic system, such as FA [127,128,129].

## 7. Future Perspectives

### 7.1. HSC Generation from iPSCs

The production of iPSCs has fundamental requirements, such as reprogramming factors, culture conditions, target tissue, and biological assays to validate the pluripotency potential of developing cells [130]. Furthermore, iPSCs must be differentiated into the desired cells; in hereditary anemias, the desired cells are hematopoietic stem cells (HSCs)/hematopoietic progenitor and stem cells (HPSCs).

Before iPSCs can be used for blood disorder treatment, they must be differentiated into HSCs [131]. The introduction of the HoxB4 transcription factor may trigger the differentiation of iPSCs into functional HSCs [132]. In the natural development of HSCs from embryonic stem cells, HSCs originate from the primitive streak of the embryo, where “primitive” hematopoiesis occurs. However, the capability of iPSC-derived HSCs for inducing hematopoiesis in the adult host is yet to be proven, such as the production of HSCs with a normal function that are able to generate erythrocytes that function optimally for oxygen delivery in postnatal life [133].

A study showed that candidate HSCs derived from human iPSCs failed to fully reconstitute and engraft irradiated adult recipients [134]. The use of the HoxB4 transcription factor was the only successful strategy for transforming embryonic stem cells (ESCs) into HSCs with long-term efficiency in vivo [135]. Hoxb4 was utilized to produce iPSC-derived engraftable hematopoietic cells, which were effective for the management of SCA in an SCA mouse model [131]. The ectopic expression of HoxB4 resulted in abnormal myeloid/lymphoid ratios in mice and leukemogenesis in dogs and monkeys, suggesting that iPSC-derived HSCs produced using this method are not clinically relevant [135]. Therefore, further research is required to assess the intrinsic genotoxicity related to reprogramming.

Another method for inducing HSC differentiation from iPSCs involves Lhx2 transduction and a two-step in vitro differentiation procedure (lineage-directed differentiation induction and selective amplification). The result revealed reductions in tumor formation in vitro and in vivo [136]. The transduction of Lhx2 into iPSCs resulted in the robust differentiation into c-Kit+/Sca-1+/Lineage (KSL) cells in vitro. The induction frequency into KSL cells using Lhx2 transduction was superior to that using Hoxb4. Moreover, the transplantation of Lhx2-transduced hematopoietic cells into lethally irradiated mice showed multilineage repopulation of hematopoietic cells after 4 months. Furthermore, no teratoma developed 4 months after the transplantation of Lhx2-transduced HSC-like cells derived from iPSCs. This result suggests that the two-step differentiation procedure could substantially eradicate remnants of iPSCs, and it reduced the risk of residual iPSC-induced tumorigenesis [136]. Various other methods of generating HSCs/HPSCs/hemangioblasts are described in Table 3.

### 7.2. Strategies for Gene Editing of HSCs

Through genome engineering, adjustments are made to the sequence of genomic DNA. Gene (and genome) editing is a genome engineering approach, which includes DNA repair through the insertion of site-precise alterations into genomic DNA. Gene editing is well-known form of genome editing directed only on genes [148].

Several strategies can be applied for gene editing in HSCs. First, ribonucleoprotein (RNP)-based editing of human HSPCs is a potential therapy for improving or curing hematopoiesis-related disorders [149]. Second, gene-editing endonuclease (GEEN) technology can be applied. The following are required: TALENs, CRISPR/Cas9, ZFNs, and meganucleases. GEEN technology is associated with several challenges in terms of delivery, immune tolerance, editing efficiency, cytotoxicity, and off-target editing. To restrict off-target editing, some strategies can be utilized, such as preventing the establishment of off-target sites in the human genome and increasing the specificity of each TALEN DNA-binding domain [150].

TALENs are engineered and programmable nucleases that cleave and bind to specific DNA sequences. TALENs are composed of a nonspecific DNA cleavage domain fused to a customizable sequence-specific DNA-binding domain to create double-strand breaks (DSBs) [151]. TALENs have benefits such as low off-target editing, low cytotoxicity, and high specificity [152]. Their limitations include difficulty in delivering them because of their large size, the long time required to build TALENs, and the probable immune response [150].

CRISPR-Cas9 is an RNA-guided gene editing tool with advantages such as easy use, cost effectiveness, and flexibility. It has various modes of delivery, such as plasmid DNA, mRNA, and protein, with advantages and disadvantages. Given its specificity and simplicity, CRISPR/Cas9 is a powerful tool for DNA editing in biological research; it is useful for developing animal disease models, validating disease targets, facilitating genetic engineering in crops and farm animals, understanding and identifying mechanisms of genetic diseases and disorders, enabling meticulous epigenetic studies, and studying protein evolution in mammalian cells [153,154,155], and it is being applied in an ongoing clinical trial for gene editing of autologous HSPCs to cure thalassemia (phase I/II: NCT03655678).

The advantages of using plasmid DNA to deliver CRISPR/Cas9 are its low cost and high stability, and its disadvantages are low efficiency, delayed onset, and integration risk. The RNA-guided CRISPR/Cas9 method has the advantage of no insertional mutagenesis, but the disadvantage of poor stability. The protein-based method also has advantages such as a high editing efficiency, no insertional mutagenesis, low off-target effects, low immunogenicity, rapid onset, and transient duration, and its disadvantages are a high cost and contamination with bacterial endotoxin [156,157,158].

In the CRISPR-Cas9 method, RNA-guided Cas9 endonuclease might correct the mutation in genes by DNA insertion or deletion, and by transcriptional repression or activation, and multiplex targeting is possible through the changing of 20 nucleotides in RNA. Because of its simplicity, CRISPR/Cas9 automation can be adapted for various in vitro and in vivo studies, and has been utilized in studies assessing gene mechanisms and functions and for transcriptional studies, disease design, generation of animal models of diverse cancers, and evaluation of novel therapeutic strategies; however, the development of clinical management strategies using CRISPR/Cas9 is challenging. For treating genetic disorders, cardiovascular diseases, carcinogenesis, and chronic diseases (such as human chronic myeloid leukemia), advances are required in CRISPR/Cas9 genome editing for improving genome editing instruments; guiding DNA repair mechanisms; efficiently engineering immune cells and oncolytic viruses; ensuring thorough and continual oncogene knockout; enabling the delivery and packaging of the tools to the designed cells, organs, and organisms; and evaluating their safety, efficacy, and efficiency [159,160,161,162]. Several clinical trials involving CRISPR/Cas9 have been conducted on β-thalassemia, such as NCT03728322 (phase I, which targeted the HBB gene in 12 participants) and NCT03655678 (phase I/II, which targeted the BCL11A gene in 45 participants), as well as on SCD, such as NCT03745287 (phase I/II, which targeted the *BCL11A* gene in 45 participants).

ZFNs are constructed to generate a DNA DSB at the target locus. Therefore, using ZFNs, gene targeting is increased by 100–10,000-fold by stimulating the cellular DNA repair pathways. These gene-targeting devices are developed by fusing engineered zinc finger (ZF) DNA-binding domains to a nonspecific nuclease domain. These targetable DNA cleavage reagents consist of two monomer subunits. Each subunit circumscribes three ZFs, which identify the FokI endonuclease domain and nine base pairs in the full target site. ZFNs are similar to TALENs, as they depend on the FokI domain for their nuclease activity [163,164,165]. Some drawbacks of ZFNs are multiple-gene targeting, context dependence, and difficulty in their design; thus, they are not widely used in microbial genetic engineering.

Researchers have established novel customizable nucleases to generate DSBs for therapeutic genome modification. Such engineered endonucleases are classified into redesigned homing endonucleases and ZFNs. ZFNs comprise an engineered Cys2-His2 ZF domain integrated to a nuclease domain of the type IIS restriction enzyme FokI. The DNA-binding ZF domain causes the binding of the nonspecific FokI cleavage domain to a definite DNA target site in this arrangement. The initiation of a DSB is contingent upon the dimerization of ZFNs, as the FokI cleavage domain is enzymatically active only as a dimer [166,167,168].

Meganucleases (also called homing endonucleases) are one of four endonucleases used for genome engineering (the other three types are ZFNs, CRISPR/Cas9, and TALE nucleases). Meganucleases can be distinguished based on their structural motifs and sequences, such as LAGLIDADG, HNH, His-Cys box, PD-(D/E)XK, and GIY-YIG. LAGLIDADG proteins are the most well studied. Meganucleases facilitate “paragon” GT (i.e., the valid and real reversion of deleterious mutations). However, it has drawbacks; the efficacy of correction declines as the span from the primary DNA DSB increases. Moreover, if sequences are located on heterologous chromosomes or on separate homologous chromosomes, the recombination efficiency is considerably reduced; thus, this method is unsuitable for therapeutic use [169,170,171,172].

### 7.3. Risk Mitigation of Engineered Cell Therapy

In ex vivo GT, viral vector utilization is associated with various safety issues, including genotoxicity. Vector-mediated genotoxicity mainly develops from cellular protooncogene upregulation through promoter activation, promoter insertion, or gene transcript truncation, and adjoining gene associated enhancer activation. Vector-mediated genotoxicity can be prevented by carefully selecting the virus, target cell type, and unification target site, as vectors may have certain integration profiles that are cell specific. Nonviral factors, such as disease, dose, and patient age, can influence the virus potential for genotoxicity. Therefore, a clinical trial and test model intervention and the careful selection the patients are paramount to ensure safety and efficacy. Viral vectors with fewer hazards of insertional mutagenesis have been used, such as vectors that target microRNA, enhancer-blocking insulators, and self-inactivating (SIN) vectors. However, insertional mutagenesis still persists despite these efforts [173].

Another issue in HSC gene editing is clonal fitness. Clonal selection of HSCs was explained by clonal hematopoiesis of indeterminate potential (CHIP); these cells have a somatic mutation that might increase fitness, and the mutation is caused by various environmental factors, such as inflammation, aging, and therapy exposure. Mutated HSCs with increased fitness may advantageously engraft and propagate in the hematopoietic compartment after radiotherapy or chemotherapy. However, highly increased fitness may cause hematopoietic malignancy [174]. Therefore, a balance in fitness should be obtained to support engraftment and propagation of HSCs in the hematopoietic compartment and to avoid malignancy. A study showed that HSC fitness could be impaired by the ablation of the kinase ataxia telangiectasia mutated (ATM), a master regulator of the DNA damage response [175].

HSC gene editing may correct a disease-causing mutated gene, but may also cause off-target editing that is undesirable, which may lead to various unpredictable results. In gene editing using CRISPR/Cas9, the sequences of guide RNAs (gRNAs) are homologous to the intended on-target regions, and gRNAs attach to on-target sites, but may also recognize off-target regions that may have up to six mismatches [176]. Off-target regions with less mismatches influences cleavage and binding [177]. As it contains a particular RNA sequence, gRNA guides editing by Cas nuclease and aids in the identification of the target DNA region [178]. Moreover, gRNA enables the knocking out of genes through the CRISPR/Cas system, and corrects defective genes with appropriate templates [179].

Off-target regions are categorized into three main types. The first category involves additional areas at the 5′-NGG-3′ end (i.e., PAMs) that possess mismatches or substitutions [180]. The second category involves areas at different 5′-NGG-3′ sequences (i.e., PAMs) that consist of deletions (indels) and/or insertions; the areas resemble target DNA or gRNA spacer regions [181]. The DNA or RNA forms a minuscule bulge with leftover nucleotides. They strengthen Cas9 action. The off-target actions at these sites are frequently stronger than the on-target actions [182]. The third category involves the fragmenting of sequences with dissimilar 5′-NAG-3′ (i.e., PAM) regions [183]. Furthermore, there are two categories of off-target effects by CRISPR. The first is anticipated off-target effects in genomic areas that have a high sequence similarity with the target. The second is unexpected off-target effects in genomic areas that are not connected to the target [184]. In addition to prospective off-target effects, chromosomal rearrangement can occur because of DSBs generated through gene editing [185]. Consequently, sequential karyotype analysis is paramount to identify any chromosomal aberrations after gene editing [117].

Several approaches can be used to discover off-target effects in the edited genome: BLESS, CIRCLE-seq, ChIP-dCas9, ChIP-seq, Digenome-seq, DISCOVER-Seq, EndoV-seq, Multiplex Digenome-seq, fluorescent in situ hybridization (FISH), GOTI, GUIDE-seq, IDLV, LAM-HTGTS, T7E1 assay, whole exome sequencing, whole genome sequencing, deep sequencing, SITE-Seq, and VIVO [182,186,187,188]. Furthermore, various algorithms can reveal off-target effects: the ensemble learning method, CRISPR-GE, CRISPR-PLANT v2, Cas-OFFinder, CCTop, CHOPCHOP, CHOPCHOP v2, CFD score, CRISPOR, CROP-IT, CT-Finder, Feng Zhang lab’s Target Finder, MD, and PEM-seq [189,190,191].

To reduce off-target effects, the CRISPR/Cas9 method, which involves using the RNP complex, comprising a single guide RNA (sgRNA) and Cas9 protein, is promising as it has fewer off-target effects. Various methods have been established to deliver RNP into targeted cells [192]. Moreover, appropriate experimental design, careful target selection, and appropriate sg RNA design may highly reduce off-target effects.

### 7.4. Clinical Perspective

We present new insights into therapy for the most common hereditary anemia types: SCD, FA, G6PD deficiency, and thalassemia. Current disease-modifying therapies for SCD include hydrocycarbamide (also called hydroxycarbamide), which increases the expression of fetal Hb, which is a type of Hb that cannot be polymerized, as well as blood transfusion and HCT [12].

To date, many treatment approaches have been discovered, and some are still in clinical trials, including drugs inhibiting hemoglobin polymerization (hydroxyurea, 2,2-dimethylbutyrate, INCB059872, metformin; panobinostat, pomalidomide, and THU-decitabine), drugs reducing erythrocyte dehydration (clotrimazole and senicapoc), drugs reducing inflammation (canakinumab, corticosteroids, intravenous immunoglobulin, and NKTT120), drugs reducing abnormal cell adhesion (crizanlizumab, propanolol, and rivipansel), drugs reducing hypercoagulability (apixaban, rivaroxaban, and sevuparin), drugs reducing platelet activation (piroxicam, prasugrel, and ticagrelor), and drugs reducing oxidative stress (l-glutamine and *N*-acetylcysteine) [193]. However, curative treatments for SCD are HSCT and GT, which correct patients’ own HSCs, and these HSCs are then infused back to the patients [194].

HSCT is an important therapeutic option for patients with SCD [195] because HSCT can establish donor-derived erythropoiesis and, more importantly, stabilize or restore the function of affected organs in patients with SCD when performed in time [194,195,196]. In allogenic transplantation, donor availability is a major limitation; one of three donor sources are required for transplantation (matched siblings, haplo-identical family member, or an UCB or matched unrelated donor [MUD]) [197,198,199,200]. Recently, to overcome this limitation, genome editing through autologous transplantation using the patients’ own cells has provided promising results. The genetic defect in sickle HSPCs can be corrected through several approaches, namely gene addition using LV-based strategies for the correction of sickle cell causing mutation and induction of HbF [114,194].

Various challenges prevail in genome editing, such as off-target effects on DNA and RNA. The use of more efficient enzymes and the short-term use of genome editing instruments can limit off-target effects [201,202,203]. Some clinical trials have provided promising results, and no genotoxicity secondary to LVs has been reported, but the main challenge has been to maintain the myeloid donor chimerism above the 20% threshold. Clinical trials using ZFNs, TALENs, and CRISPR-Cas9 for gene editing have also provided promising results, but these gene editing methods are in the early phases of development [194,204,205].

For FA, which is a DNA repair disorder in any of the 22 FA genes, gene-corrected HSCs have promising outcomes as a treatment alternative to HSCT, which is the preferred therapy for BMF in FA patients. Gene-corrected FA HSCs may progressively ensure normal hematopoiesis in FA patients treated with GT, even in the absence of conditioning. In a recent study, Rio et al. [206] showed that although the number of HSCs is low in patients with FA, the proliferation of HSPCs was observed in patients with FA and led to mosaicism, which suggested that the infusion of low numbers of gene-corrected HSCs might be sufficient for their engraftment after autologous transplantation.

So far, no clinical trial has investigated stem cell or GT treatments for G6PD deficiency. A previous study showed that human G6PD deficiency could be stabilized by HSC transduction using the retroviral vector harboring human G6PD cDNA. Patients with severe chronic nonspherocytic hemolytic anemia related to G6PD deficiency might be treated with allogenic BMT or GT, but no study has investigated this approach. People with G6PD deficiency should avoid drugs and chemicals that can cause oxidative stress, and they should avoid broad beans, which are known to be the trigger of hemolysis in G6PD deficiency patients. Identification and discontinuation of the precipitating agents are important in order to avoid hemolysis [207].

Treatment for thalassemia depends on the clinical manifestation. Mild forms of thalassemia do not require specific treatment, but for moderate to severe thalassemia, treatment includes blood transfusion for correction of anemia, the suppression of erythropoiesis, and the inhibition of gastrointestinal iron absorption. Splenectomy is recommended if the patient has indications such as symptomatic splenomegaly with the risk of splenic rupture, and an iron chelating agent is required to prevent iron overload as a consequence of regular transfusion [208].

Some novel therapeutic approaches are being developed for purposes such as the correction of the globin chain imbalance, targeting ineffective erythropoiesis, and modulating iron metabolism. For the correction of the globin chain imbalance, BMT might be an option for patients with TDT to restore the capability of functional Hb production. This HSCT should be conducted early on in transfusion-dependent patients with an MSD, before iron overload and subsequent tissue damage, but this option has some limitations in terms of the availability of a suitable donor, patient fitness, and procedure-related toxicity. To overcome these limitations, clinical trials using gene editing strategies to reactivate HbF were conducted, but transfusion independence could not be achieved for most thalassemia β^0^/β^0^. Another trial using the CRISPR/Cas9 strategy to edit patients’ HSCs is being conducted by Sangamo Therapeutics (NCT03432364), and the preliminary results showed successful gene editing of peripheral white blood cells collected from the thalassemia β^0^/β^0^ patient. The preliminary results showed an increase in fetal Hb levels and stable total Hb values around 9 g/dL at 50 days from the infusion [101].

The genetic defect in HSCs can be corrected by the transfer of a normal gene using a suitable vector such as LV, or through homologous recombination. Patient-specific iPSCs are a promising alternative therapy for β-thalassemia in the future, but the related animal experiments are still limited; further study is still needed to confirm this result [112].

## 8. Conclusions

Our review shows that the main cause of sickle cell disease, FA, G6PD deficiency, and thalassemia is genetic mutation, but they have different pathogeneses that all lead to anemia. Anemia in sickle cell disease and G6PD deficiency is due to hemolysis, while in FA and thalassemia it is due to impaired red blood cell production. Gene-corrected HSCT has promising outcomes for sickle cell disease, FA, and thalassemia, and can overcome the limitations of the source of allogeneic BMT.

## Figures and Tables

**Table 1:** Current clinical trials for sickle cell disease (SCD) treatments [45,47,48,49,50,51,52].

Trial Phase(s)	Drug Compounds (and Explanation)
Phase I RCT	Metformin, Aes-103, SCD-101 (NCT02380079), and NKTT120 (NCT01783691)
Phase I	Ambrisentan (NCT02712346) Decitabine + tetrahydrouridine or THU (NCT01685515) Plerixafor mobilization and apheresis (NCT03226691–multicenter study) Citrulline * (NCT02314689, NCT02697240) Zileuton (NCT01136941–SAOL) Panobinostat or LBH589 (NCT01245179) INCB059872 (NCT03132324)
Phase I/II RCT	Voxelotor or GBT440 (NCT02850406)
Phase I/II	Arginine (NCT02447874–open-label randomized crossover design), rivipansel or GMI-1070 (NCT00911495–SAOL, NCT01119833–RDBPC, and NCT02187003–RDBPC), omega-3 fatty acids (NCT02947100–SAOL), *N*-acetylcysteine (NCT01800526–SAOL), and Simvastatin (NCT0050802–SAOL, NCT01702246–SAOL)
Phase II RCT	Crizanlizumab, rivipansel, intravenous Ig, or IVIG (NCT01757418–RDBPC), dalteparin, sevuparin, eptifibatide, prasugrel, haptoglobin, oral or intravenous nitrite, inhaled nitric oxide, hemopexin/haptoglobin, MP4CO, various antioxidants, canakinumab, montelukast, and simvastatin.
Phase II	Atorvastatin (NCT01732718), Arginine (NCT01796678–RDBPC, NCT02536170–RDBPC, and NCT00004412–open-label randomized design), Mometasone (NCT02061202), Montelukast (NCT01960413), Omega-3 fatty acids (NCT02973360–RDBPC), AMD 3100 or Mozobil (plerixafor) (NCT00075335), Riociguat (NCT02633397–RDBPC), and IW-1701 (NCT03285178–RDBPC).
Phase III RCT	Arginine, senicapoc, tinzaparin, ticagrelor, rivipansel (GMI-1070), crizanlizumab (NCT03814746), and antioxidants.
Phase III	Glutamine (NCT01179217–RDBPC), Omega-3 fatty acids (NCT02525107, NCT02604368), and *N*-acetylcysteine (NCT01849016–RDBPC).
FDA-approved	l-glutamine, hydroxyurea, crizanlizumab, or SEG101 (NCT1895361–RDBPC, NCT03264989–SAOL, and NCT03474965–SAOL).
Under investigation	TLR4 inhibition, DNAse-1, anakinra, and vitamin D.

* Study drug administered during acute VOC, SAOL: single-arm open-label, RDBPC: randomized double-blind placebo-controlled.

**Table 2:** Classification of thalassemia (adapted from [72]).

Classification	Types		Transfusions	Explanation
Thalassemia minor	α-thalassemia trait β-thalassemia trait Homozygous HbE/C HbE or C trait		Seldom required	
Thalassemia intermedia	β-thalassemia intermedia HbC/β-thalassemia	Mild HbE/β-thalassemia, HbH with β-thalassemia trait	Occasionally required	Non-transfusion-dependent thalassemia: (NTDT)
		Deletional HbH, nondeletional HbH, moderate HbE/β-thalassemia EF Bart’s disease, AE Bart’s disease	Intermittently required	
Thalassemia major	Nondeletional HbH Survived Hb Bart’s hydrops β-thalassemia major Severe HbE/β-thalassemia		Regular, lifelong transfusion required	Transfusion-dependent thalassemia (TDT)

**Table 3:** Methods of hematopoietic stem cells (HSCs) generation from induced pluripotent stem cells (iPSCs).

Cell Source(s)	Differentiated into	Methods	Engraftment Assay Result	References
Human iPSCs	HSPCs	Monolayer method to generate HPSCs and transduction of HPSCs using MLL-AF4	B and T cells and myeloid engraftment at the 8th week	[9,137]
Mouse iPSCs	HSPCs	Transduction of iPSCs using Gfi1b, c-Fos, and Gata2 followed by teratoma formation in vivo in mice. HSPCs were taken from bone marrow	B and T cells and myeloid engraftment at the 16th week	[9,138,139]
Human iPSCs	HSPCs	Teratoma formation in vivo by subcutaneous transplantation with or without OP9 feeder cells and cytokines. HSPCs were taken from the bone marrow	B and T cells and myeloid engraftment at the 4th and 12th weeks	[9,139]
Monkey iPSCs	CD34+ HSCs	Embryoid body formation followed by culture on E4ORF1-transduced HUC–derived primary endothelial cells, and sorting of CD34+ HSCs	Myeloid, lymphoid, and erythroid engraftment at the 12th and 16th weeks	[9,139,140]
Mouse iPSCs	T cells	Transduction using Runx1 and Hoxa9 followed by embryoid body formation, sorting of hemogenic endothelial cells, and culture on OP9-DL1 cells	T-cell engraftment at the 4th and 6th weeks	[9,139,141]
Human iPSCs	CD34+ CD45+ HSCs	Embryoid body formation using BMP4 and cytokines followed by sorting of CD34+ and CD45+ cells, and transduction using HOXA9, ERG, RORA, SOX4, and MYB to increase engraftment capacity	Erythroid and myeloid engraftment at the 4th and 5th weeks	[9,139,142]
Human iPSCs	CD34+ CD45+ HPSCs	iPSC alone or co injection with OP9, OP9W3a, or OP9D to induce teratoma in mice followed by HPSC isolation from teratoma	Human HPSC engraftment in the spleen and lymph node at the 8th week	[9,139,143]
Mouse and human iPSCs	HSCs	iPSCs co injected with OP9 stromal cells into mice to form teratomas. HSCs were taken from the bone marrow	From mouse iPSCs blood cells engraftment at the 4th and 12th weeks From Human iPSCs erythrocytes and CD3+ T cells engraftment at 8 weeks	[9,139,144]
Human iPSCs	Hemangioblasts	Culture on the MEF feeder layer	Not assayed	[9,139,145]
Human iPSCs	Hemangioblasts	3D culture using Matrigel-coated microcarrier.	Not assayed	[9,146,147]

GFi1B: Growth factor independent 1B transcriptional repressor, a protein-coding gene. E4ORF1: Control protein E4 open reading frames (ORFs). HUC: Human umbilical cord. ERG: (E-twenty-six)-related gene, a critical factor protecting HSCs from differentiation. Hoxa9: Homeobox protein Hox-A9. MEF: Mouse embryonic fibroblast. MYB: A gene encoding various transcription factors that contain typical DNA binding motifs, a master regulator of hematopoietic system development and function. NSG: NOD scid γ, NOD.Cg-Prkdc^scid^ Il2rg^tm1Wjl^/SzJ. OP9-DL1: Delta-like 1 is expressed by the OP9 stromal cell line. RORA: Receptor-related orphan receptor α. RUNX1: RUNX family transcription factor 1. SOX4: SRY-related high-mobility group box 4.

## Data Availability

Not applicable.
